# Fully integrated colorimetric sensor based on transparency substrate for salbutamol determination

**DOI:** 10.1016/j.mex.2022.101913

**Published:** 2022-11-05

**Authors:** Atchara Lomae, Sudkate Chaiyo, Orawon Chailapakul, Weena Siangproh, Janjira Panchompoo

**Affiliations:** a Electrochemistry and Optical Spectroscopy Center of Excellence, Department of Chemistry, Faculty of Science, Chulalongkorn University, Pathumwan, Bangkok 10330, Thailand; b The Institute of Biotechnology and Genetic Engineering, Chulalongkorn University, Pathumwan, Bangkok 10330, Thailand; c Department of Chemistry, Faculty of Science, Srinakharinwirot University, Sukhumvit 23, Wattana, Bangkok 10110, Thailand

**Keywords:** Salbutamol, Potassium permanganate, Transparency-based analytical device, Colorimetric detection, Redox reaction

## Abstract

A facile colorimetric method based on a typical redox reaction was first developed for the determination of salbutamol (SAL) using a low-cost and portable transparency-based analytical device (TAD). The TAD was simply fabricated by wax-printing onto a transparent polymer-based substrate to create the hydrophobic barriers and the colorimetric reaction zones where the color changes could be easily observed with the naked eye. Potassium permanganate (KMnO_4_), a common oxidizing agent, was deliberately used as a colorimetric reagent for SAL. Once SAL reacted with KMnO_4_ in the acidified system, it could undergo oxidation and the color of KMnO_4_ subsequently changed from light pink to orange. The color change corresponding to the SAL concentration could be clearly observed at the TAD sensor. In addition, the reaction color could be recorded using a digital camera and then analyzed by ImageJ for quantitative analysis. Under the optimized conditions, the developed method together with the TAD sensor exhibited high efficiency for SAL determination with linearity ranging from 0.5 to 40 mg·L^−1^ and a limit of detection (LOD) of 0.05 mg·L^−1^.

• This proposed TAD-based colorimetric method using permanganate as color reagent showed excellent performance in SAL detection with good accuracy and high precision.

## Highlights

• Facile colorimetric method for the determination of salbutamol based on a simple redox reaction using permanganate as reagent was first investigated at the transparency-based analytical device (TAD).
• The colorimetric TAD sensor was in-lab constructed using a transparent PET-based substrate, showing good compatibility with the permanganate reagent which could result in a vivid color change, clearly observed by the naked eye.
• This proposed colorimetric TAD sensor was successfully applied for salbutamol determination in real drug samples with good accuracy and satisfactory recovery, highlighting the potential capability of this TAD-based colorimetric sensor in pharmaceutical application.

## Graphical Abstract

**Figure:**
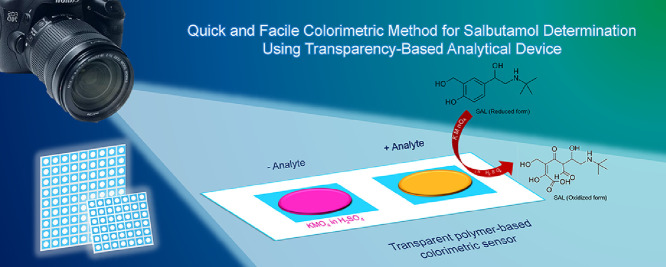

Specifications table

**Table:** 

Subject Area:	Chemistry
More specific subject area:	Development of the transparency-based analytical device (TAD) for colorimetric determination of salbutamol
Method name:	Colorimetric assay using a transparency-based analytical device for salbutamol determination
Name and reference of original method:	Simultaneous determination of β-agonists by UHPLC coupled with electrochemical detection based on palladium nanoparticles modified BDD electrode, Journal of Electroanalytical Chemistry, 840 (2019) 439-448.
Resource availability:	https://doi.org/10.1016/j.jelechem.2019.04.003

## Introduction

Salbutamol (SAL), which belongs to a class of ß_2_-adrenergic agonists, is commonly used as bronchodilator for the treatment of human chronic obstructive pulmonary disease and asthma [1,2]. Besides, SAL is generally employed in veterinary medication [3] as it can induce relaxation of smooth muscle and decrease systemic vascular resistance [4]. However, high doses of SAL have anabolic-like effects (*e.g.* promoting protein production for muscle build-up and enhancing strength performance) which are sometimes used illegally by athletes in sports. According to the World Anti-Doping Agency (WADA), the concentration of SAL greater than 1000 ng/mL (equal to 3 µM) found in urine literally indicates the doping behavior in athletes [5], [6], [7], [8]. Moreover, SAL is the most common ß_2_-agonist used in animal feed as growth promoter and meat leaner, enhancing the transformation of nutritive body fat to more muscle in livestock [1], [2], [3]. However, SAL used in animals for growth promotion would readily accumulate in their tissues and organs and could directly be transferred to humans through the food chain (*e.g.* consumption of animal products), causing serious effects on human health in numerous symptoms including palpitation, tremors and nervousness [1,2]. The use of SAL as growth promoter has generally been banned in many countries, especially in China and the European Union [9,10]. Therefore, the development of a reliable and sensitive analytical device and method for determination of SAL with quick identification and confirmation is crucially needed to control the drug abuse for both growth promotion and sports-doping.

According to the literature, various analytical techniques have previously been developed for SAL determination, including UHPLC-ECD [11], HPLC-UV/Vis [12], LC-MS [13], LC-MS/MS [10], UPLC-MS/MS [5], CE [14,15] and immunoassay [16]. Although these methods are comparatively precise and sensitive, they inevitably have some disadvantages, such as time-consuming process, complicated procedure, expensive instrumentation, and unsuitable for quick screening test and on-site applications [17,18]. Consequently, colorimetry which has become highly attractive for qualitative and quantitative analysis seems to be the potential method for rapid detection of SAL, due to its simplicity, straightforward signal readout, minimum sample and reagent consumption, and inexpensive apparatus. Considering the colorimetric readout, UV-vis spectrophotometer has been reported to be the traditional tool for colorimetry [19,20], but the instrument itself is still somewhat sophisticated and unsatisfactory for on-site applications. Hence a digital camera or a camera phone accompanying with the image processing application has been simply used as an alternative device for colorimetric detection with potential applications for point-of-care and on-site testing [21], [22], [23]. Moreover, the microfluidic technology has been currently utilized for miniaturization of various portable devices, making them fitting for field-based testing applications. Generally, papers have been widely used as substrates in this technology, to fabricate various types of “paper-based analytical devices (µPADs)”. Since first development in 2007, the µPAD has experienced rapid growth over the past decade because of their affordable, simple and portable properties [24,25]. Due to the sustainability and superior mechanical properties of cellulose, the µPADs possess the capillary-driven fluid flow, allowing the sample and the reagent to flow through the hydrophilic fibrous area within the device without the requirement of external instruments [26,27]. With this aspect, the µPADs have been developed in several patterns and in different types of platforms depending on their applications such as the storage of liquid reagents [28,29], the stop flow devices [30] and the channels for sample pretreatment [31]. Although the µPAD has numerous advantages, its chemical resistance is quite low which may limit the use of µPAD in the corrosive media. Other polymer-based materials such as plastic commercial films (both transparent and opaque) which are made of either polyvinyl chloride (PVC) or polyethylene terephthalate (PET) have also become of great interest as substrate materials due to their chemical inertness, physical robustness, excellent flexibility and high resistance to water and moisture, resulting in the long term stability and durability, compared to the µPADs [32].

In this work, the transparent polymer-based material, namely PET has been first employed as substrate for the fabrication of a portable gadget, so-called “transparency PET-based analytical device (TAD)” for colorimetric determination of SAL. Unlike the µPADs, the developed TAD could be simply manufactured by wax printing onto the PET sheet, generating the hydrophobic regions and the transparent colorimetric detection areas where a vivid color change could be obviously seen (owning to the high transparency of PET). The obtained TAD could then be used compactly in association with a simple colorimetric method in which the acidified potassium permanganate (KMnO_4_) was basically employed as reagent for facile colorimetric determination of SAL. Typically, potassium permanganate is a well-known strong oxidizing agent that could quickly oxidize SAL in acidic system, while the permanganate itself would simultaneously underdo reduction, resulting in the color change (from light pink to light orange) which could be clearly observed either by the naked eye for qualitative screening test, or by a digital camera together with an image processing software for quantitative analysis. Subsequently, the proposed colorimetric device and method could be potentially applied for SAL detection in bronchodilator drug samples with comparable results to those labelled on the medicine, demonstrating a proof of concept for quality control in pharmaceutical industry.

## Chemicals and Materials

Salbutamol sulfate was purchased from Sigma-Aldrich (Missouri, USA). Potassium permanganate (KMnO_4_) used as colorimetric reagent was obtained from Carlo Erba Reagents (Chaussée du Vexin, French). To determine the exact concentration of potassium permanganate solution; once prepared, it was standardized with sodium oxalate (Na_2_C_2_O_4_) which was bought form J.T. Baker chemical company (Loughborough, UK). Sulfuric acid (H_2_SO_4_) was gained from Merck (reagent grade 95-97%, Gernsheim, Germany). Milli-Q water form Millipore (R ≥ 18.2 MΩ cm) was used throughout experiments.

### Design and fabrication of TADs

A wax-printing technique, which has been reported to be a simple, fast and low-cost method for the fabrication of various µPADs [33], [34], [35], was deliberately used in this work to create the TAD. Generally, the device pattern designing was performed by Adobe illustrator software, and the wax-pattern layout was then printed onto the transparency-based PET sheet (bought from local store in Bangkok, Thailand) using the wax printer (Xerox ColorQube 8570, Japan) in order to create the hydrophobic barrier and the transparent colorimetric reaction zone with diameter of 0.6 cm. Note that the color of hydrophobic wax-pattern was selected to be complementary to the colorimetric reaction region.

### Colorimetric detection of SAL on the TAD

Potassium permanganate was simply used as a colorimetric reagent for SAL detection. The measurement was performed as follows: 10 µL of KMnO_4_ were directly dropped onto the transparency PET-based device at the transparent reaction zone, following by 20 µL of H_2_SO_4_ and 5 µL of standard SAL (with varying concentrations), respectively. Next, the final volume of solution mixture was adjusted to 40 µL using Milli-Q water. The color change (from pale pink to varying shades of tinted orange, depending on the concentration of SAL) at the reaction zone could subsequently be observed by the naked eye within the optimized reaction time of 11 min, applicable to qualitative measurement. As for quantitative analysis, the image of the resulting color of the solution mixture on the TAD was thoroughly recorded by a digital camera (Cannon EOS 1000 D1, Japan) in a light-control box, and the color intensity of the sample solution was then measured by ImageJ software using gray scale mode. Note that in order to reduce the light reflection in the light-control box, the stencil paper was used as a filter for reducing the scattered light by fixing a distance at 2 cm between the light source and the stencil paper in the light-control box. Finally, a calibration curve showing the relationship between color intensity and concentration of SAL could be constructed.

### Analysis of SAL in pharmaceutical samples

Various brands of pharmaceutical tablets containing SAL (purchased from the local drug store) were used for validation of the proposed colorimetric method with the developed TAD. Firstly, the tablets were weighed out in order to determine the average weight per tablet, and they were ground using a mortar and pestle. Then, a certain amount of sample powder was weighed once again into a 100-mL volumetric flask. After that, the drug sample was extracted with 50 mL of Milli-Q water for 1 h in a shaker, followed by a sonication in an ultrasonic bath for 10 min. The final volume of sample solution was subsequently adjusted to 100 mL using Milli-Q water. The obtained sample solution would then be filtered through a 0.45 µM polytetrafluoroethylene (PTFE) syringe filter to remove undissolved binder, prior to use in the experiment [6,36,37].

Regarding the recovery study, it should be noted that the standard SAL was spiked into the sample solution before the extraction in three different concentrations of 2.5, 10.0 and 25.0 ppm, respectively. The percent recovery of SAL added to the drug sample could then be calculated from these spiked samples.

### Results and discussion

**Fig. 1:**
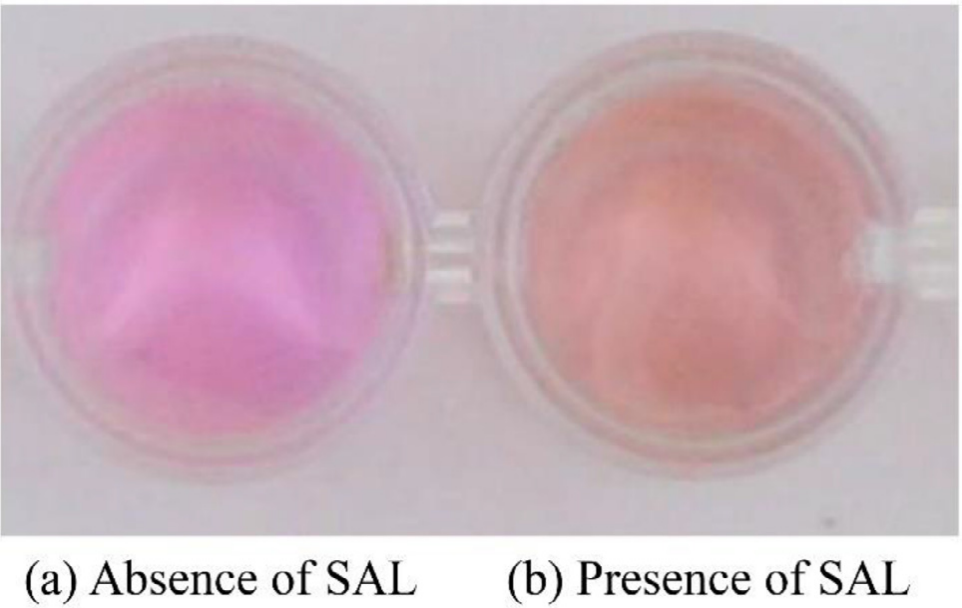
The reaction solution of 0.15 mM KMnO_4_ in 0.5 mM H_2_SO_4_ in the absence (a) and presence (b) of 10 ppm SAL, and the color change from light pink (a) to pale orange (b) could be clearly observed in a 96-well plate.

In this work, SAL was first determined using potassium permanganate (KMnO_4_), a very common oxidant, as a colorimetric reagent. Once SAL reacted with permanganate in an acidified system at room temperature, the color of permanganate would simply change from light pink to pale orange which could be clearly observed, as shown in Fig 1.

The reaction of SAL and potassium permanganate which caused the color change could be simply explained by a redox process and the corresponding standard half-cell potentials (E^0^) of both half reactions. Typically, permanganate has been very well known to be a powerful oxidizing agent, and the oxidation behaviour of SAL has also been extensively reported in the literature. Therefore, in this colorimetric measurement, SAL which was colorless would get oxidized with its standard half-cell potential of −0.78 V *vs* SHE [8], as shown in Eq (1), whereas the pale pink permanganate ion would simultaneously undergo reduction in mildly acidic condition with the reported standard reduction potential of +1.68 V *vs* SHE [38] to form the brownish manganese(IV) oxide (MnO_2_), leading to the color change from light pink to orange, as shown in Eq (2). The standard cell potential ($E_{\text{cell}}^{0}$) of this redox reaction could be calculated and it was found to be +2.46 V *vs* SHE, confirming the spontaneous reaction between SAL and permanganate ion. 

**Fig. 2:**
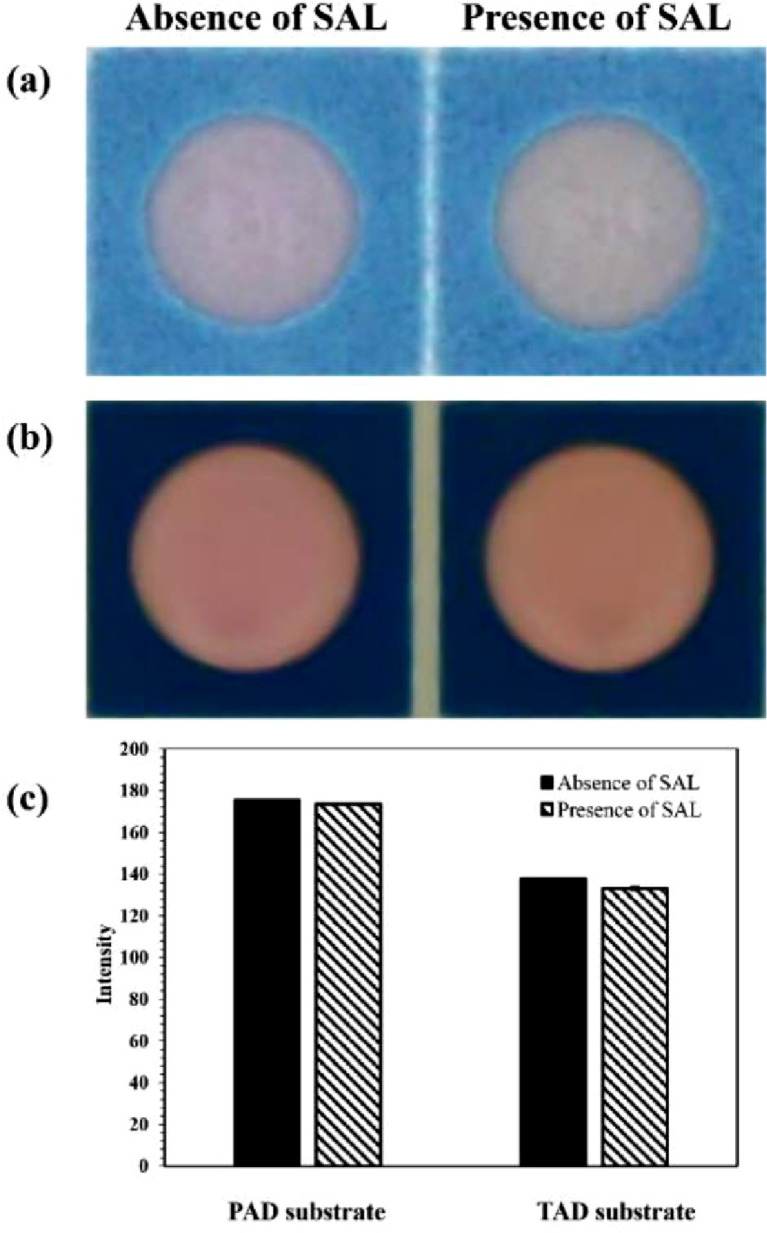
Color change observed on the PAD (a) and the TAD (b) with the reaction solution of 0.15 mM KMnO_4_ in 0.5 mM H_2_SO_4_ in the absence and presence of 10 ppm SAL, as well as the plot (c) showing the color intensity (in gray scale mode) of the resulting redox reaction on the PAD and the TAD substrates (all data was obtained from three repetitive experiments).

As for the development of portable device, the fabrication of the miniaturized colorimetric devices for SAL detection has been thoroughly investigated by wax-printing onto two types of substrates, including paper and PET sheet which resulted in the PAD and the TAD, respectively. The results demonstrated that the TAD seemed to be more compatible with the proposed redox-based colorimetric detecting method, giving more intense and well steady color which could be precisely observed, compared to the PAD, as shown in Fig 2. It can be seen that the pink color of the permanganate itself on the PAD seemed already too faded at the beginning, and when SAL was subsequently added to react with the permanganate reagent, the color change became less intense and unstable (Fig 2(a)). This could be possibly due to the side reaction between the oxidizing permanganate agent and the cellulose-based paper where the C_6_-OH unit of cellulose could also get oxidized to C_6_-OOH by permanganate at a high reaction rate [39,40], resulting in the unclear and inconsistent color of the reagent on the PAD [41]. Conversely, no such side reaction of the permanganate reagent could occur at the PET-based transparency sheet [42,43], resulting in more obvious color distinction that could be clearly observed at this transparency platform (Fig. 2(b)), compared to the PAD.

In addition, the color intensity (in gray scale mode) resulting from the proposed redox reaction on the PAD and the TAD was briefly compared. Fig 2(c) shows that the larger difference in the color change intensity (ΔI = 4.65) could be observed on the TAD-based substrate, compared to that obtained from the PAD-based device (ΔI = 1.99), demonstrating that the TAD would typically be more suitable for the colorimetric detection of SAL using permanganate as the reagent to observe the color change.

Therefore, the transparent PET sheet was chosen as substrate material for the fabrication of colorimetric TAD sensor as it was chemically inert towards the permanganate ion, and it also provided a vivid color change for SAL determination.

The experimental conditions for the colorimetric determination of SAL on the TAD have been next investigated. Since the colorimetric detection was performed under the acidified system using permanganate as the regent, the corresponding parameters, including type of acid, concentration of acid, concentration of permanganate reagent, reaction time, and reaction temperature were therefore optimized. In this study, a representative SAL concentration of 10 ppm was used constantly in the optimization as it would cause a well detectable color change, and the color intensity of the reacting solution before and after SAL addition was measured and reported as *I_blank_* and *I_sample_*, respectively. The intensity value of the color change (∆I) according to the redox reaction between permanganate and SAL could then be quantitatively calculated by subtracting the blank solution from the sample response (∆I *= I_sample_ – I_blank_*).

**Fig. 3:**
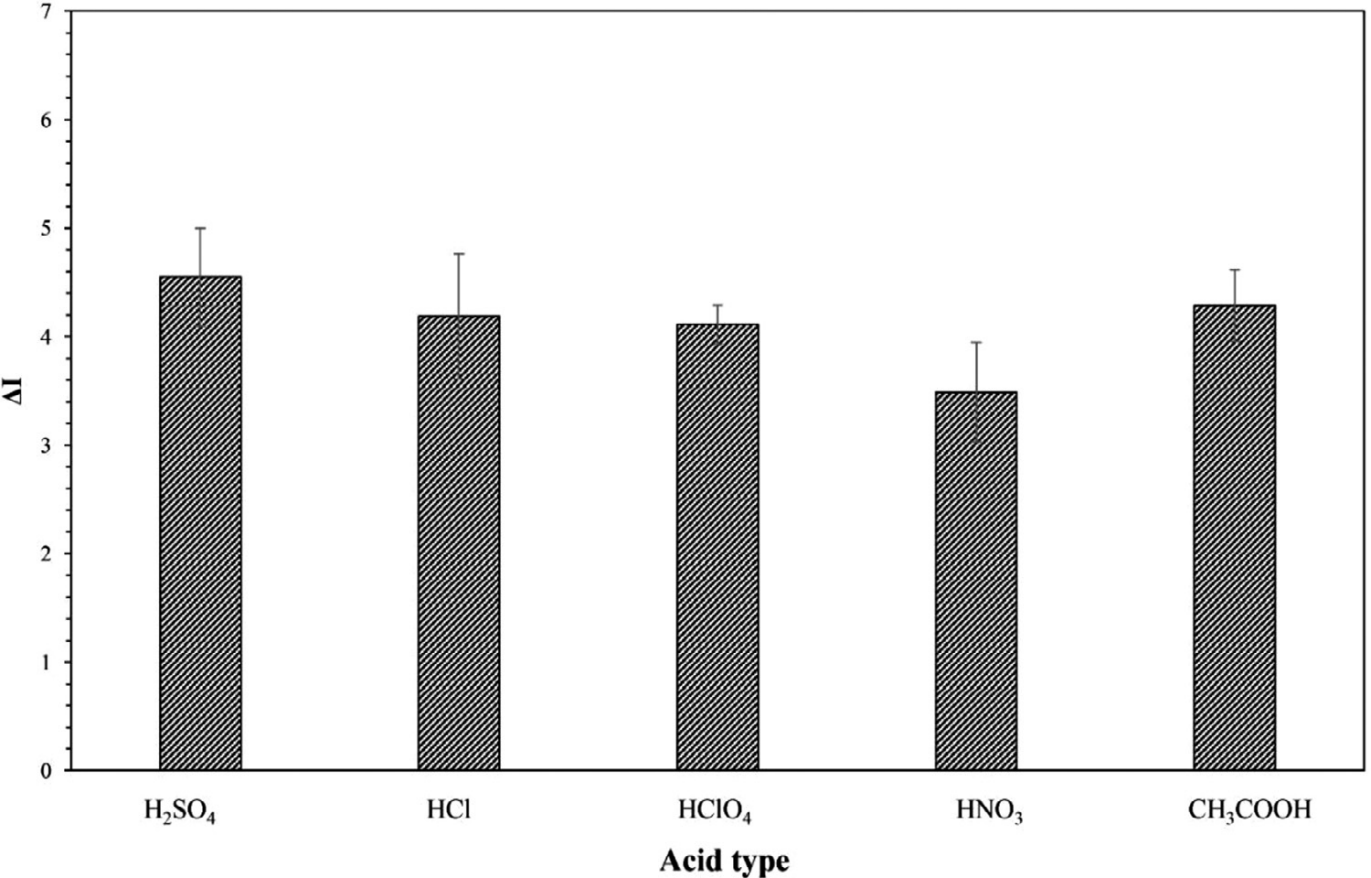
Effects of acid type on the colorimetric determination of SAL using permanganate in acidic medium as reagent.

The influence of acid type on the colorimetric detection of SAL was first examined using various strong and weak acids, including sulfuric acid (H_2_SO_4_), hydrochloric acid (HCl), perchloric acid (HClO_4_), nitric acid (HNO_3_), and acetic acid (CH_3_COOH) at the same concentration of 1 mM. Fig 3 shows the plots of sample's color intensity change (∆I) against various types of acid used in this study. As clearly seen form the results, different types of acid, either strong or weak ones, did not seem to have a significant effect on the color change of permanganate when reacted with SAL. However, it should be noted that the redox reaction between permanganate and SAL had to be done in acidic medium as the reduction process generally required additional protons, as illustrated in Eq (1). Therefore, sulfuric acid was chosen as acid species used further in this work, according to the previous study reported by J. Huclová, et.al. [7] Moreover, sulfuric acid would dissociate into sulfate ion which was the common ion already present in the system from the dissolution of salbutamol sulfate (C_13_H_21_NO_3_·0.5H_2_SO_4_) used as standard SAL reagent.

**Fig. 4:**
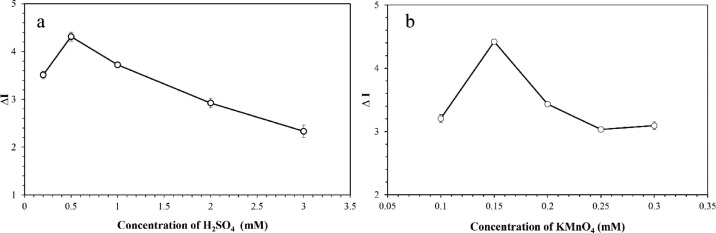
Plots showing the effects of experimental parameters, including (a) concentration of H_2_SO_4_, and (b) concentration of KMnO_4_ on the colorimetric determination of SAL.

The concentration of sulfuric acid that might affect the color change intensity in the colorimetric measurement of SAL was next examined over a range of 0.2−3.0 mM, and the obtained results were displayed in Fig 4(a). It could be clearly observed that the color change intensity (∆I) of the reacting solution increased with increasing concentration of sulfuric acid from 0.2 to 0.5 mM. After that, the ∆I intensity decreased constantly with increasing concentrations of sulfuric acid up to 3.0 mM. As discussed earlier that the reduction of permanganate would generally occur under slightly acidic condition (*cf.* Eq (1) indicating the four-proton-accepting reaction), the mild concentration of sulfuric acid of 0.5 mM likely seemed to be more favored by that redox reaction, showing the highest color change intensity. When high concentrations of sulfuric acid (with several more protons as well) were present in the system, the permanganate ion could further undergo a five-electron and eight-proton transfer reduction to form the colorless Mn^2+^ [38], leading to the drop in orange-ish color intensity, as observed in this study. Therefore, the optimal concentration of sulfuric acid highly capable of detecting SAL was found to be 0.5 mM.

The effects of permanganate concentration used as the colorimetric reagent was subsequently investigated in a concentration range of 0.1−0.3 mM, and the corresponding results were shown in Fig 4(b). Typically, the ∆I value increased with increasing concentration of permanganate ion up to 0.15 mM. When the permanganate concentration was above 0.15 mM, the color change intensity got declined instantly. Since high concentration of permanganate would give an intense purple color which could not visibly fade away during the redox reaction, the color change of this intensely purple permanganate could not be clearly observed during the reaction with SAL. Consequently, the concentration of 0.15 mM KMnO_4_, possessing a pale pink color was selected as an optimal condition for further measurements.

**Fig. 5:**
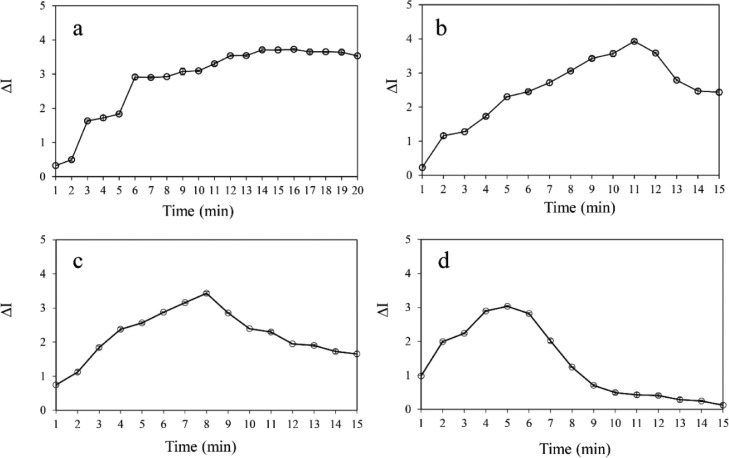
Effects of reaction time (studied within 20 min) and temperature at (a) 25°C, (b) 30°C, (c) 40°C and (d) 50°C on the colorimetric detection of SAL.

The reaction time and temperature that would play an important role in the reaction kinetics have been also investigated. Briefly, the effects of reaction time and temperature were studied within 1−20 min at 25°C, and 1−15 min at 30, 40 and 50°C, respectively and each experiment was performed in triplicate. Fig 5 shows the color change intensity (∆I) observed over a range of time at increasing temperatures. The highest color change intensity could be achieved within 14, 11, 8 and 5 min at 25, 30, 40 and 50°C, respectively, indicating that the maximum reaction had already developed. The results clearly demonstrated that the reaction rate increased with increasing temperature. However, at the temperatures above 30°C (*cf.* Fig 5(c) and (d)), the sample's color intensity observed at the largest values of ∆I got somewhat decreased, resulting in lowering the sensitivity of the detection by *ca.* 18.2% when compared to the highest ∆I obtained at 30°C (*cf.* Fig 5(b)). This is possibly due to the gradual generation of Mn(II), a reducing species obtained from a side reaction of permanganate taking place at higher temperature, as shown in Eq (3). When Mn(II) was present in the reaction system, it could further react with the permanganate agent to form Mn(III) as a colorless product, as shown in Eq (4).

(3) MnO_4_^−^ + 8H^+^ + 5e^−^ → Mn^2+^ + 4H_2_O

(4) 4Mn^2+^ (orange color) + MnO_4_^−^ (pink color) + 8H^+^ → 5Mn^3+^ (colorless) + 4H_2_O

Generally, the side reactions involving Mn(II) and permanganate have been reported to be dominated by elevated temperature for a proper period of reaction time [44]. When the reaction temperature was over 30°C, the rapid fading of the color change intensity could be observed, likely due to the side reaction between Mn^2+^ (orange color) and permanganate (pink color) toward the colorless color (Mn^3+^), resulting in a decrease in color change intensity observed at the largest values of ∆I. Therefore, the reaction temperature of 30°C together with the 11 min reaction time were chosen as the optimal conditions for the quick and sensitive determination of SAL.

**Fig. 6:**
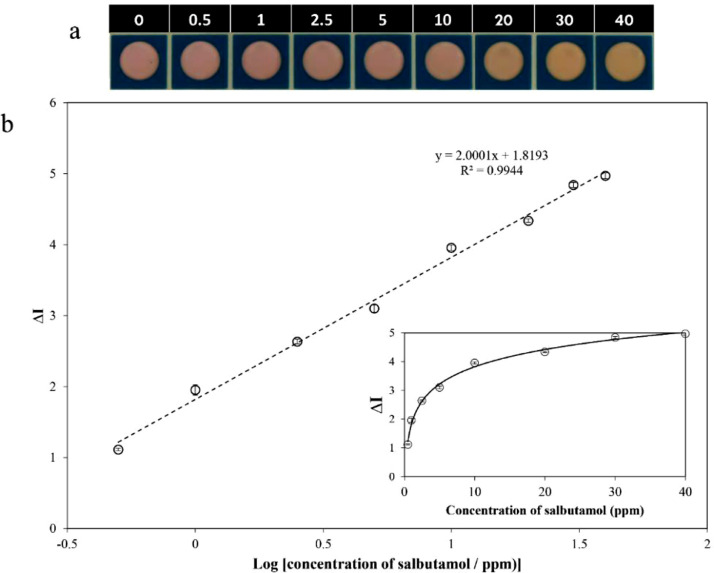
(a) Corresponding images of the colorimetric detection of SAL at various concentrations ranging from 0−40 ppm on the TAD colorimetric sensor, and (b) Plot of linear regression analysis with the SAL concentrations of 0.5−40.0 ppm in a log scale. Inset: Plot of the average color change intensity against the concentration of SAL in a range of 0.5−40.0 ppm.

Under the optimal conditions, the performance of the proposed colorimetric method combined with the TAD was subsequently investigated for quantitative analysis of SAL. Upon the addition of SAL in the concentration ranging from 0−40 ppm, the light pink of permanganate reagent slightly changed to orange-tinted color, as shown in Fig 6(a). Basically, the experiments were performed in triplicate, and the average color change intensity (∆I) obtained was then plotted against the SAL concentration, as shown as an inset in Fig 6(b). As clearly seen in the inset in Fig 6(b), the color intensity values of the reacting solution increased steadily but not linearly with the increase of SAL concentration. When the color change intensity (∆I) was plotted with respect to the log scale of SAL concentration, as shown in Fig 6(b), a linear response of SAL detection could be obtained in the range of 0 to 40 ppm at 30°C with a correlation coefficient of 0.9944. Furthermore, the limits of detection (LOD) and quantification (LOQ) could be calculated using 3σ method (where σ is the standard deviation of the blank solution), and found to be 0.05 and 0.17 ppm, respectively.

## Conclusion

A transparent PET-based analytical device (TAD), which is inexpensive, portable, and simply manufactured, suitable for colorimetric application was first developed for facile qualitative and quantitative detection of SAL. The TAD was rapidly and easily fabricated by wax-printing method and the colorimetric detection of SAL was simply performed based on the redox reaction of the oxidizing permanganate reagent and the reducing SAL sample. In the presence of SAL, the color of permanganate in acidic medium would visibly change from light pink to pale orange at 30°C within 11 min reaction time, which could be clearly observed by the naked eye. The proposed colorimetric TAD sensor showed high performance towards SAL measurement with low detection limit of 0.05 ppm. Additionally, this method was successfully applied for determination of SAL in several drug samples where the acceptable relative errors of less than 9%, the satisfied recoveries of 81.00 – 108.87%, and the good RSD of 1.32 – 7.53% could be thoroughly obtained, highlighting that this colorimetric method coupled with the TAD sensor was highly capable of detecting SAL with high accuracy and precision.

## Supplementary material *and/or* Additional information: Colorimetric determination of SAL in real samples

In order to validate the developed colorimetric method and the corresponding TAD sensor, the determination of SAL in a range of commercially available drug samples has been performed using permanganate as the colorimetric reagent spotting on the proposed transparent PET-based device. A recovery study was also carried out using an external standard method. Generally, it was found that the experimental values for various drug samples were in good agreement with those labelled on the medicine with relative errors less than 8.8%, demonstrating good accuracy for quantitative analysis, as shown in Table 1. In addition, the %recoveries and %RSDs of the spiked drug samples were found to be in the range of 81.00 − 108.87% and 1.32 − 7.53%, respectively, as presented in Table 2. The results typically showed satisfactory recoveries with high precision, as well as indicating a proof of concept of the potential capability of the developed colorimetric method and TAD sensor in pharmaceutical application.

**Table 1:** Colorimetric determination of SAL in real drug samples using permanganate as oxidizing agent at the transparent PET-based sensor.

Samples	Salbutamol content		% Relative error
	Labeled value (mg / tablet)	Contents found (mg / tablet)	
Sample 1	2	2.18 ± 0.06	8.8
Sample 2	2	2.05 ± 0.21	2.7
Sample 3	2	1.85 ± 0.11	7.3
Sample 4	4	4.30 ± 0.36	7.4
Sample 5	4	4.13 ± 0.20	3.3
Sample 6	4	4.19 ± 0.17	4.7

**Table 2:** Recovery study for SAL determination in the spiked drug samples (n=3).

Sample	Spiked (ppm)	Found (ppm) Mean ± SD	Recovery (%) Mean ± SD	RSD (%)
Sample 1	0	10.89 ± 0.29	-	-
	2.5	12.98 ± 0.17	83.60 ± 6.93	1.33
	10	21.55 ± 0.57	106.60 ± 5.72	2.65
	25	35.52 ± 2.20	98.53 ± 8.79	6.19
Sample 2	0	10.27 ± 1.06	-	-
	2.5	12.98 ± 0.34	108.53 ± 13.63	2.62
	10	18.76 ± 0.25	84.93 ± 2.48	1.32
	25	37.45 ± 0.99	108.72 ± 3.95	2.64
Sample 3	0	9.27 ± 0.53	-	-
	2.5	11.31 ± 0.15	81.47± 6.00	1.33
	10	18.37 ± 1.38	81.00 ± 13.83	7.53
	25	33.68 ± 2.40	97.64 ± 9.59	7.12
Sample 4	0	10.74 ± 0.91	-	-
	2.5	13.39 ± 0.18	105.87 ± 7.16	6.67
	10	20.26 ± 0.27	95.23 ± 2.71	2.85
	25	37.45 ± 0.99	106.84 ± 3.95	3.70
Sample 5	0	10.33 ± 0.50	-	-
	2.5	12.98 ± 0.17	106.00 ± 6.93	6.54
	10	21.22 ± 0.28	108.87 ± 2.83	2.60
	25	36.05 ± 0.99	102.89 ± 3.97	3.86
Sample 6	0	10.47 ± 0.42	-	-
	2.5	12.78 ± 0.17	92.53 ± 6.70	7.24
	10	19.65 ± 0.52	91.80 ± 5.20	5.66
	25	36.90 ± 1.93	105.73 ± 7.74	7.32

## Declaration of Competing Interest

The authors declare that they have no known competing financial interests or personal relationships that could have appeared to influence the work reported in this paper.

## Data Availability

Data will be made available on request.
